# Supplementary material for: Evolution of multicellular life cycles under costly fragmentation
Source: PLoS Comput Biol. 2020 Nov 19;16(11):e1008406. doi: 10.1371/journal.pcbi.1008406 (PMC7714367; doi:10.1371/journal.pcbi.1008406)
Supplement: S1 Text — Life cycles competition. (PDF) [file pcbi.1008406.s001.pdf]

Appendix 1. Life cycles competition

In the result of population dynamics described above, some life cycles become more abundant, while others go extinct. The outcome of this life cycle competition is independent on he resource limitation parameter (K) and is exactly the same as in the linear model without resource limitation at all ($K \to \infty$).

To show this, consider the whole population describe by values $x_i^\kappa(t)$ - the number of groups of size i in the lineage executing life cycle κ. We can decompose this value into a form

$$x_i^\kappa(t) = X(t) f^\kappa(t) \rho_i^\kappa(t), \tag{1}$$

where $X(t)$ is the total number of units in a population, $f^\kappa(t)$ is the fraction of units following the life cycle κ, and $\rho_i^\kappa(t)$ is the fraction of them, which have a size i. Naturally,

$$\sum_\kappa f^\kappa(t) = 1, \tag{2}$$

$$\sum_i \rho_i^\kappa(t) = 1. \tag{3}$$

The quantity $f^\kappa(t)$ characterizes the evolutionary success of a life cycle, and we are generally interested in its dynamics.

In Eqs. (3) , the terms responsible for units growth, death, and fragmentation are linear with respect to x_i, and therefore, these equations can be represented as

$$\frac{d}{dt} x_i = \sum_j A_{ij} x_j - \frac{1}{K} X x_i, \tag{4}$$

where A_{ij} is some constant matrix. In particular, this equation is valid independently for each life cycle κ.

Combining Eqs. (4) and (1), we get

$$\frac{d}{dt}(X f^\kappa \rho_i^\kappa) = X f^\kappa \sum_j A_{ij} \rho_j^\kappa - \frac{1}{K} X^2 f^\kappa \rho_i^\kappa. \tag{5}$$

Summation over all units sizes i in Eq. (5) results with

$$\frac{d}{dt}(X f^\kappa) = X f^\kappa \sum_{i,j} A_{ij} \rho_j^\kappa - \frac{1}{K} X^2 f^\kappa. \tag{6}$$

And after rearranging the terms

$$\frac{d}{dt}f^{\kappa} = f^{\kappa}\sum_{i,j} A_{ij}\rho_j^{\kappa} - \frac{1}{K}Xf^{\kappa} - f^{\kappa}\frac{1}{X}\frac{d}{dt}X. \tag{7}$$

There, summation over all life cycles provides

$$\frac{1}{X}\frac{d}{dt}X = \sum_{i,j,\kappa} A_{ij}\rho_j^{\kappa}f^{\kappa} - \frac{1}{K}X. \tag{8}$$

Plugging Eq. (8) back into Eq. (7), we finally obtain

$$\frac{d}{dt}f^{\kappa} = f^{\kappa}\left(\sum_{i,j} A_{ij}\rho_j^{\kappa} - \sum_{i,j,\mu} A_{ij}\rho^{\mu}f^{\mu}\right). \tag{9}$$

The dynamics of life cycles competition explicitly given by Eq. (9) is independent on the severity of the resources limitation K and the total number of units X. The competition of life cycles has the same outcome in the model without resource competition at all ($K \to \infty$).
