# Supplementary material for: Evolution of multicellular life cycles under costly fragmentation
Source: PLoS Comput Biol. 2020 Nov 19;16(11):e1008406. doi: 10.1371/journal.pcbi.1008406 (PMC7714367; doi:10.1371/journal.pcbi.1008406)
Supplement: S2 Text — Linear model of life cycles evolution. (PDF) [file pcbi.1008406.s002.pdf]

Appendix 2. Linear model of life cycles evolution

Without resource competition, the population dynamics is governed by a system of equations

$$\frac{d}{dt}x_1 = -b_1x_1 - d_1x_1 + \pi_1(\kappa)mb'_mx_m \tag{1a}$$

$$\frac{d}{dt}x_i = -ib_ix_i + (i-1)b_{i-1}x_{i-1} - d_ix_i + \pi_i(\kappa)mb'_mx_m \quad \text{for} \quad 1 < i < m \tag{1b}$$

$$\frac{d}{dt}x_m = -mb'_mx_m + (m-1)b_{m-1}x_{m-1} - d'_mx_m + \pi_m(\kappa)mb'_mx_m. \tag{1c}$$

The equation system Eq. (1) is linear with respect to x_i. Thus, it can be written as:

$$\frac{d}{dt}\mathbf{x} = A\mathbf{x}, \tag{2}$$

where $\mathbf{x} = (x_1, x_2, \cdots, x_m)^T$ and the matrix A is

$$A = \begin{pmatrix} -b_1 - d_1 & 0 & 0 & \cdots & \pi_1(\kappa)mb'_m \\ b_1 & -2b_2 - d_2 & 0 & \cdots & \pi_2(\kappa)mb'_m \\ 0 & 2b_2 & -3b_3 - d_3 & \cdots & \pi_3(\kappa)mb'_m \\ 0 & 0 & 3b_3 & \cdots & \pi_4(\kappa)mb'_m \\ \vdots & \vdots & \vdots & \ddots & \vdots \\ 0 & 0 & 0 & \cdots & \pi_m(\kappa)mb'_m - mb'_m - d'_m \end{pmatrix} \tag{3}$$

In the long run, the solution of Eq. (2) converges to that of an exponentially growing population with a stable distribution, i.e.,

$$\lim_{t\to\infty} \mathbf{x}(t) = e^{\lambda t}\mathbf{w}. \tag{4}$$

The leading eigenvalue λ gives the total population growth rate, and its associated right eigenvector $\mathbf{w} = (w_1, \ldots, w_m)$ gives the stable distribution of unit sizes.

The leading eigenvalue determines the evolutionary success of a population. In the competition of populations utilizing different life cycles (and hence different λ), each of them will grow independently of the others. Eventually, the population with the largest growth rate will outcompete the others. Thus, natural selection would promote the life cycle that provides the largest λ. We call this the evolutionarily optimal life cycle.
