# Supplementary material for: Evolution of multicellular life cycles under costly fragmentation
Source: PLoS Comput Biol. 2020 Nov 19;16(11):e1008406. doi: 10.1371/journal.pcbi.1008406 (PMC7714367; doi:10.1371/journal.pcbi.1008406)
Supplement: S3 Text — Forbidden fragmentation modes. (PDF) [file pcbi.1008406.s003.pdf]

Appendix 3.

Characteristic equation for an arbitrary life cycle

Consider a life cycle in which the number of cells in a unit increases until the maturity size m is reached and once the next cell is born, a unit fragments according to a partition κ of $j' \leq m+1$. The corresponding projection matrix is an $m \times m$ matrix of the form

$$A = \begin{pmatrix} -b_1 - d_1 & 0 & 0 & 0 & \cdots & mb'_m\pi_1(\kappa) \\ b_1 & -2b_2 - d_2 & 0 & 0 & \cdots & mb'_m\pi_2(\kappa) \\ 0 & 2b_2 & -3b_3 - d_3 & 0 & \cdots & mb'_m\pi_3(\kappa) \\ 0 & 0 & \ddots & \ddots & \ddots & \vdots \\ 0 & 0 & 0 & \cdots & (m-1)b_{m-1} & mb'_m\pi_m(\kappa) - mb'_m - d'_m \end{pmatrix}. \tag{1}$$

The population growth rate is given by the leading eigenvalue λ_1 of A, i.e., the largest solution of the characteristic equation

$$\det\left(A - \lambda\mathbf{I}\right) = 0. \tag{2}$$

By using a Laplace expansion along the last column of $A - \lambda\mathbf{I}$, we can rewrite the left hand side of the above expression (i.e., the characteristic polynomial of A) as

$$\begin{aligned} \det\left(A - \lambda\mathbf{I}\right) &= \sum_{i=1}^{m-1}(-1)^{i+m} mb'_m\pi_i(\kappa)M_{i,m} + (-1)^{2m}\left(mb'_m\pi_m(\kappa) - mb'_m - d'_m - \lambda\right)M_{m,m} \\ &= \sum_{i=1}^{m}(-1)^{i+m} mb'_m\pi_i(\kappa)M_{i,m} - \left(mb'_m + d'_m + \lambda\right)M_{m,m} \end{aligned} \tag{3}$$

where $M_{i,m}$ is the (i,m) minor of $A - \lambda\mathbf{I}$. For all $i = 1, \ldots, m$, the minor $M_{i,m}$ is the determinant of a block diagonal matrix, and hence equal to the product of the determinants of the diagonal blocks. Moreover, each diagonal block is either a lower triangular or an upper triangular matrix, whose determinant is given by the product of the elements in their main

diagonals. We can then write

$$M_{i,m} = \prod_{j=1}^{i-1} (-jb_j - d_j - \lambda) \prod_{j=i}^{m-1} jb_j. \tag{4}$$

Substituting Eqs. (3) and (4) into Eq. (2), switching the order of the two terms and simplifying, we obtain

$$\begin{aligned} &- (-1)^{m-1} (mb'_m + d'_m + \lambda) \prod_{j=1}^{m-1} (jb_j + d_j + \lambda) \\ &+ (-1)^{m-1} \sum_{i=1}^{m} mb'_m \pi_i(\kappa) \prod_{j=1}^{i-1} (jb_j + d_j + \lambda) \prod_{j=i}^{m-1} jb_j = 0. \end{aligned}$$

Dividing both sides by

$$(-1)^m \prod_{j=1}^{m} jb_j,$$

we get

$$\begin{aligned} &\frac{mb'_m + d'_m + \lambda}{mb_m} \prod_{j=1}^{m-1} \left(1 + \frac{d_j + \lambda}{jb_j}\right) \\ &- \sum_{i=1}^{m} \frac{b'_m}{b_m} \pi_i(\kappa) \prod_{j=1}^{i-1} \left(1 + \frac{d_j + \lambda}{jb_j}\right) = 0. \end{aligned}$$

We rewrite the factor in front of the product in the first line as

$$\frac{mb'_m + d'_m + \lambda}{mb_m} = \left(1 + \frac{d_m + \lambda}{mb_m}\right) + \frac{m(b'_m - b_m) + d'_m - d_m}{mb_m}.$$

Thus,

$$\begin{aligned} &\prod_{j=1}^{m} \left(1 + \frac{d_j + \lambda}{jb_j}\right) + \frac{m(b'_m - b_m) + d'_m - d_m}{mb_m} \prod_{j=1}^{m-1} \left(1 + \frac{d_j + \lambda}{jb_j}\right) \\ &- \frac{b'_m}{b_m} \sum_{i=1}^{m} \pi_i(\kappa) \prod_{j=1}^{i-1} \left(1 + \frac{d_j + \lambda}{jb_j}\right) = 0. \end{aligned}$$

Simplifying this, we finally obtain that the characteristic equation (2) can be written as

$$F_{m+1}(\lambda) + \Delta_m F_m(\lambda) - \frac{b'_m}{b_m} \sum_{i=1}^{m} \pi_i(\kappa) F_i(\lambda) = 0, \tag{5}$$

where

$$F_i(\lambda) = \prod_{j=1}^{i-1} \left(1 + \frac{d_j + \lambda}{jb_j}\right). \tag{6}$$

and

$$\Delta_i = \frac{i(b'_i - b_i) + d'_i - d_i}{ib_i}. \tag{7}$$

Note that two transformations preserve Eq. (5):

$$d_i \to d_i - r, \qquad d' \to d' - r, \qquad \lambda_1 \to \lambda_1 + r, \qquad r \leq \min(\mathbf{d}), \tag{8}$$

and

$$\mathbf{d} \to s\mathbf{d}, \qquad \mathbf{b} \to s\mathbf{b},$$
$$b' \to sb', \qquad d' \to sd', \qquad \lambda_1 \to s\lambda_1, \qquad s > 0.$$

These transformations allow us to set $b_1 = 1$ and $\min(\mathbf{d}) = 0$ without loss of generality.

Forbidden fragmentation modes

For any environment, for any combination of the fragmentation delay, risk and fixed loss, the fragmentation mode having two different subsets of offspring with the same combined size is dominated. To prove this, we use approach similar to one used in Appendix E in [1]. Consider positive integers m, j, k such that $m + 1 \geq 2j + k$, two partitions of j, τ_1 and τ_2, such that $\tau_1 \neq \tau_2$, and an arbitrary partition ϕ of k, and the following three deterministic fragmentation modes:

1. $\kappa_1 = \tau_1 + \tau_2 + \phi$ – the partition of $2j + k \leq m + 1$, whereby a unit fragments upon the increment of size from m to $m + 1$ into a number of offspring given by partitions τ_1, τ_2, and ϕ.

2. $\kappa_2 = \tau_1 + \tau_1 + \phi$ – the partition of $2j + k \leq m + 1$, whereby a unit fragments upon the increment of size from m to $m + 1$ into a number of offspring given by two partitions τ_1 and one partition ϕ.

3. $\kappa_3 = \tau_2 + \tau_2 + \phi$ – the partition of $2j + k \leq m + 1$, whereby a unit fragments upon the increment of size from m to $m + 1$ into a number of offspring given by two partitions τ_2 and one partition ϕ.

Denoting by $\lambda(\kappa_i)$ the leading eigenvalue of the projection matrix induced by fragmentation mode κ_i, we can show that, for any environment, either $\lambda(\kappa_1) \leq \lambda(\kappa_2)$ or $\lambda(\kappa_1) \leq \lambda(\kappa_3)$ holds. Thus, a fragmentation mode with two different subsets of offspring with the same combined size is dominated by a mode where one of these subsets repeats twice, while the other one is not present.

To prove the statement above, let us define the polynomial $p_i(\lambda)$ as the left hand side of Eq. (5) with $\kappa = \kappa_i$, so that $\lambda(\kappa_i)$ is the largest root of $p_i(\lambda)$. We obtain

$$p_1(\lambda) = F_{m+1}(\lambda) + \Delta_m F_m(\lambda) - \frac{b'_m}{b_m}\left(\sum_{i=1}^{m} \pi_i(\tau_1)F_i(\lambda) + \sum_{i=1}^{m} \pi_i(\tau_2)F_i(\lambda) + \sum_{i=1}^{m} \pi_i(\phi)F_i(\lambda)\right) \tag{9a}$$

$$p_2(\lambda) = F_{m+1}(\lambda) + \Delta_m F_m(\lambda) - \frac{b'_m}{b_m}\left(2\sum_{i=1}^{m} \pi_i(\tau_1)F_i(\lambda) + \sum_{i=1}^{m} \pi_i(\phi)F_i(\lambda)\right) \tag{9b}$$

$$p_3(\lambda) = F_{m+1}(\lambda) + \Delta_m F_m(\lambda) - \frac{b'_m}{b_m}\left(2\sum_{i=1}^{m} \pi_i(\tau_2)F_i(\lambda) + \sum_{i=1}^{m} \pi_i(\phi)F_i(\lambda)\right) . \tag{9c}$$

These polynomials satisfy the following two properties. First,

$$\lim_{\lambda \to \infty} p_i(\lambda) = \infty, \tag{10}$$

as the leading coefficient of the left hand side of (5) is given by $(b_1 \cdot b_2 \cdot \ldots \cdot b_m m!)^{-1}$, which is always positive. Second,

$$p_1(\lambda) = \frac{p_2(\lambda) + p_3(\lambda)}{2}. \tag{11}$$

Since $\lambda(\kappa_1)$ is a root of $p_1(\lambda)$, evaluating Eq. (11) at $\lambda(\kappa_1)$ leads to

$$p_2(\lambda(\kappa_1)) = -p_3(\lambda(\kappa_1)).$$

Hence, one of the following three scenarios is satisfied:

(i) $p_2(\lambda(\kappa_1)) < 0 < p_3(\lambda(\kappa_1))$,

(ii) $p_2(\lambda(\kappa_1)) > 0 > p_3(\lambda(\kappa_1))$, or

(iii) $p_2(\lambda(\kappa_1)) = p_3(\lambda(\kappa_1)) = 0$.

If $p_2(\lambda(\kappa_1)) < 0 < p_3(\lambda(\kappa_1))$ due to Eq. (10) and Bolzano's theorem (if a continuous function has values of opposite sign inside an interval, then it has a root in that interval), $p_2(\lambda)$ has

a root between $\lambda(\kappa_1)$ and ∞. Therefore, $\lambda(\kappa_1) \leq \lambda(\kappa_2)$ holds, i.e. the largest root of $p_2(\lambda)$ is larger than the largest root of $p_1(\lambda)$. Next, let us focus on (ii): If $p_2(\lambda(\kappa_1)) > 0 > p_3(\lambda(\kappa_1))$, then $\lambda(\kappa_1) \leq \lambda(\kappa_3)$ holds. Finally, if $p_2(\lambda(\kappa_1)) = p_3(\lambda(\kappa_1)) = 0$, then both $\lambda(\kappa_1) \leq \lambda(\kappa_2)$ and $\lambda(\kappa_1) \leq \lambda(\kappa_3)$ hold.

We conclude that either $\lambda(\kappa_1) \leq \lambda(\kappa_2)$ or $\lambda(\kappa_1) \leq \lambda(\kappa_3)$ must hold. Thus, the life cycle corresponding to κ_1 can never lead to the highest growth rate.

References

[1] Y. Pichugin, J. Peña, P. Rainey, and A. Traulsen. Fragmentation modes and the evolution of life cycles. *PLoS Computational Biology*, 13(11):e1005860, 2017.
