# Supplementary material for: Evolution of multicellular life cycles under costly fragmentation
Source: PLoS Comput Biol. 2020 Nov 19;16(11):e1008406. doi: 10.1371/journal.pcbi.1008406 (PMC7714367; doi:10.1371/journal.pcbi.1008406)
Supplement: S4 Text — Random environments. (PDF) [file pcbi.1008406.s004.pdf]

Appendix 4. Random environments

Uncorrelated random environments

We numerically investigate the distribution of optimal life cycles on two sets of environments: random environments and random detrimental environments, which strongly disfavour multicellular units. Both sets are explored by 10000 environments generated only once and then used to assess all three scenarios: delay, risk, and loss.

In the set of random environments, each element of the division and death rates vector ($\mathbf{b}$ and $\mathbf{d}$) was sampled independently from the uniform distribution $U(0, 1)$.

Random detrimental environments

To construct an unbiased set of random detrimental environments we used a method adopted from [1]. For each environment, we initially sampled two sequences of $n = 19$ random numbers from the uniform distribution $U(0, 1)$. Then, the first sequence has been sorted in descending order to form the vector of the division rates $\mathbf{b}$ and the second sequence has been sorted in ascending order to form the vector of death rates $\mathbf{d}$. Thus, in all detrimental environments, the values of division rates monotonically decreased with the unit size, while the values of death rates monotonically increased. Therefore, life cycles that fragment at large sizes only are strongly disfavoured.

Random beneficial environments

The set of random beneficial environments was constructed in a similar way. There, for each environment, we first sampled two sequences of $n = 19$ random numbers from the uniform distribution $U(0, 1)$. Then, the first sequence has been sorted in ascending order to form the vector of the increasing division rates $\mathbf{b}$ and the second sequence has been sorted in descending order to form the vector of decreasing death rates $\mathbf{d}$. In the result, the largest possible size is the most productive and the most defended state of a multicellular unit.

Random unimodal environments

To construct the set of random unimodal environments, we again started with two sorted sequences of random numbers from the uniform distribution $U(0, 1)$. Then, to construct the division rates vector, the largest value of the first sequence was assigned to the optimal size of 10 cells. The second largest value was randomly assigned either to the position on the left (9 cells) or the right (11 cells). Then, the next largest value was randomly assigned to either of two free slots closest to 10-th position. Hence, the vector of division rates has the maximum at 10 cells and monotonically decreases in both directions away from it. The vector of death rates was constructed in the similar way, with the minimum of death rate being at the optimal size of 10 cells.

References

[1] J. Peña, B. Wu, and A. Traulsen. Ordering structured populations in multiplayer cooperation games. *Journal of the Royal Society Interface*, 13:20150881, 2016.
