# Supplementary material for: Evolution of multicellular life cycles under costly fragmentation
Source: PLoS Comput Biol. 2020 Nov 19;16(11):e1008406. doi: 10.1371/journal.pcbi.1008406 (PMC7714367; doi:10.1371/journal.pcbi.1008406)
Supplement: S5 Text — Binary fragmentation and (nearly) equal split are overrepresented in beneficial and unimodal environments. (PDF) [file pcbi.1008406.s005.pdf]

Appendix 5. Binary fragmentation and (nearly) equal split are overrepresented in beneficial and unimodal environments.

Besides environments with uncorrelated random values of birth and death rates (see Fig. 4 in the main text) and detrimental environments (see Fig. 5 in the main text), we investigated beneficial and unimodal environments. Both demonstrated patterns qualitatively similar to the environments with uncorrelated random values, see Figs. 1 and 2.

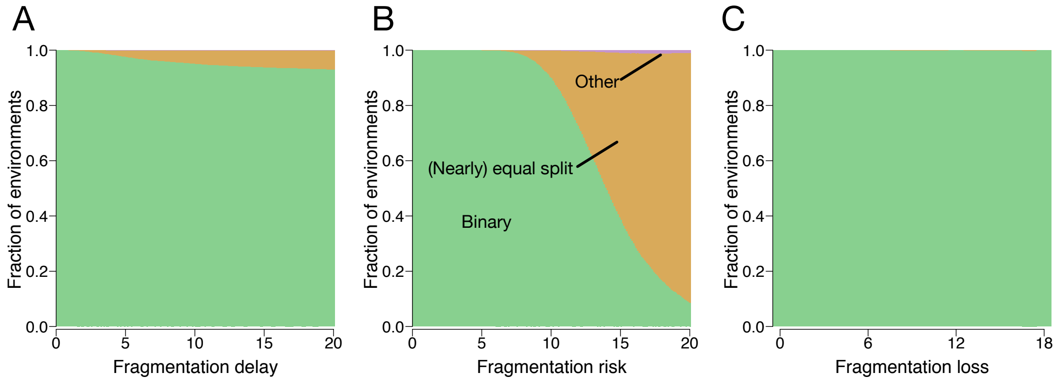

Figure 1: **Binary fragmentation and (nearly) equal split are overrepresented in beneficial environments.** In this case, fragmentation always occurs at the largest possible size. The fractions of each of four classes of life cycles under (**A**) delay, (**B**) risk, and (**C**) loss fragmentation costs.

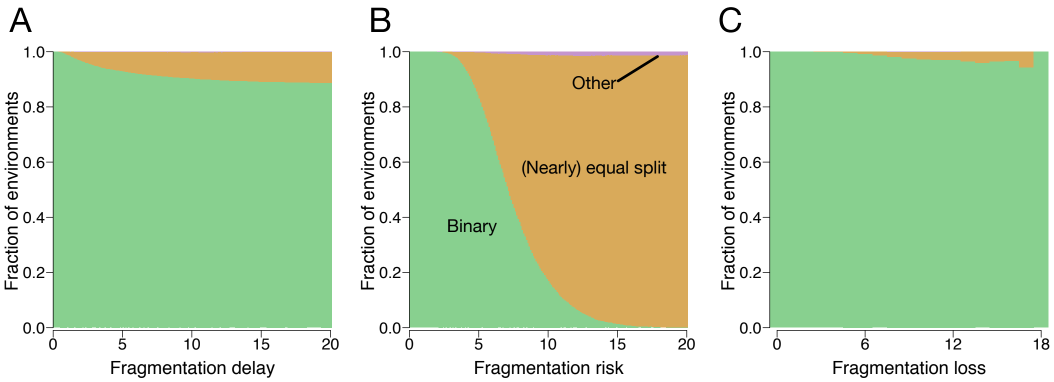

Figure 2: **Binary fragmentation and (nearly) equal split are overrepresented in unimodal environments.** The fractions of each of four classes of life cycles under (**A**) delay, (**B**) risk, and (**C**) loss fragmentation costs.
