# Supplementary material for: Evolution of multicellular life cycles under costly fragmentation
Source: PLoS Comput Biol. 2020 Nov 19;16(11):e1008406. doi: 10.1371/journal.pcbi.1008406 (PMC7714367; doi:10.1371/journal.pcbi.1008406)
Supplement: S6 Text — Only deterministic fragmentation modes can be evolutionarily optimal under any environment. (PDF) [file pcbi.1008406.s006.pdf]

Appendix 6. Only deterministic fragmentation modes can be evolutionarily optimal under any environment

Following [2], the state of the population can be described by the vector $\mathbf{x}$, where x_i denotes the abundance of units of size i. All processes changing the state vector $\mathbf{x}$ – birth, death and fragmentation – occur with a constant rate. Thus, the dynamics of the population state can be described by a set of linear differential equations or, equivalently, by a matrix differential equation

$$\dot{\mathbf{x}} = A\mathbf{x}, \tag{1}$$

where A is a projection matrix defined by demographics of the population [1]. An element $a_{i,j}$ of the projection matrix describes the rate of change of the number of units of size i caused by processes occurring with units of size j.

To construct the projection matrix elements, consider units of a certain size j. We denote by $q_{j,\kappa}$ the probability that upon the increase in size from j to $j+1$, the unit will fragment by a partition κ of $j' \leq j+1$ (where the "$\leq$" indicates that cells can be lost upon fragmentation). Among these partitions we distinguish the trivial partition of $j+1$ that corresponds to the cell division without fragmentation; we denote this by $q_{j,(j+1)}$. The combined probability of all outcomes is equal to one:

$$\sum_{\kappa} q_{j,\kappa} = 1. \tag{2}$$

For deterministic life cycles, only one partition occurs in all cell clusters in a population. Thus, for unit sizes j up to maturity size m, the trivial partition occurs with probability one ($q_{j,(j+1)} = 1$), while all other partitions have zero probability. Once a cell divides in a cluster that reached the maturity size, a certain non-trivial partition of $j' \leq m+1$ occurs with probability one. In a stochastic life cycle, more than one partition has non-zero probability at least at one unit size.

To show that stochastic life cycles are dominated by deterministic ones, we construct the projection matrix for an arbitrary stochastic life cycle. The number of cells in a unit increases by one cell at a time, thus no process can increase the size of a cluster by more than one unit

at once, so $a_{i,j} = 0$ for all $i > j + 1$. Thus, the projection matrix may contain non-zero elements only in the upper right triangle (emergence of smaller units during fragmentation), on the main diagonal (fragmentation, cell division and death of units), and on the first lower subdiagonal (increment of a unit size due to a cell division).

The first lower subdiagonal describes the rate of emergence of new larger units due to cell division without fragmentation. These rates are equal to the product of the number of cells in a unit, their division rate, and the probability the fragmentation does not occur:

$$a_{j+1,j} = jb_j q_{j,(j+1)}. \tag{3}$$

The upper right triangle of the matrix describes the emergence of new units as a result of fragmentation of larger units. For a given partition κ and given size of the newborn unit i, the rate of production of new units is equal to the product of the fragmentation rate (jb'_j), the probability to fragment according to the given partition ($q_{j,\kappa}$), and the number of units of given size produced in the act of fragmentation with this partition ($\pi_i(\kappa)$). The value of an element $a_{i,j}$ in the upper left triangle is equal to the sum of rates provided by all partitions available to clusters of size j,

$$a_{i,j} = jb'_j \sum_{\kappa} q_{j,\kappa} \pi_i(\kappa). \tag{4}$$

The main diagonal $a_{j,j}$ describes the changes in units numbers due to cell division and fragmentation as well as the death of units. The first component of $a_{j,j}$ captures that once a unit of size j increase in size or fragments, the number of units of that size decreases. The rates of decrease are equal to $jb_j q_{j,(j+1)}$ due to cell division and $jb'_j \sum_\kappa q_{j,\kappa}$ due to fragmentation. The second component is provided by a fragmentation with partition $\kappa = j+1$, which produce units of size equal to the size of parent. This leads to an increase in the number of units of size j at rate $jb'_j q_{j,j+1} \pi_j(j+1)$, where $\pi_1(1+1) = 2$ and $\pi_j(j+1) = 1$ if $j > 1$. The last component of $a_{i,i}$ comes from the death of units, which leads to a decrease in their number at rate $d_j q_{j,(j+1)} + d'_j \sum_\kappa q_{j,\kappa}$, where the first term describes the death rate in the absence of the fragmentation and the second term describes the death rate of fragmenting units.

Combined, the diagonal elements of projection matrix are

$$a_{j,j} = -jb_j q_{j,(j+1)} - jb'_j \sum_{\kappa} q_{j,\kappa} + jb'_j q_{j,j+1} \pi_j(j+1) - d_j q_{j,(j+1)} - d'_j \sum_{\kappa} q_{j,\kappa}. \quad (5)$$

All elements of the projection matrix given by Eq. (3)-(5) are linear with respect to any probability $q_{j,\kappa}$. As shown in [2], in this case the optimal life cycle is always deterministic, independent of the parameter values, such as the division and death rates and the scenario of the fragmentation cost.

References

[1] H. Caswell. *Matrix population models*. Sinauer Associates, Sunderland MA, 2nd edition, 2001.

[2] Y. Pichugin, J. Peña, P. Rainey, and A. Traulsen. Fragmentation modes and the evolution of life cycles. *PLoS Computational Biology*, 13(11):e1005860, 2017.
