# Supplementary material for: Evolution of multicellular life cycles under costly fragmentation
Source: PLoS Comput Biol. 2020 Nov 19;16(11):e1008406. doi: 10.1371/journal.pcbi.1008406 (PMC7714367; doi:10.1371/journal.pcbi.1008406)
Supplement: Attachment — Submitted filename: Reply to reviewers_05.docx [file pcbi.1008406.s007.docx]

First of all, we would like to thank the reviewers and editors for all their comments!

Reviewer #1: The authors point out that a great variety of life cycles exist in nature, although the factors affecting the evolution of life cycles and fragmentation modes are largely unknown. The authors previously found that costless fragmentation promotes the evolution of binary fragmentation modes, but fragmentation into more than two parts is common in biology. Costly fragmentation, however, is widespread in nature, and this article is on how costly fragmentation influences the evolution of life cycles.

In the model that the authors investigate, cells of an organism divide and stay together until they reach some maximum size, at which point the fragmentation mode determines how the organism divides into smaller groups of cells. The authors consider three types of fragmentation costs: delay, death risk, and cell loss. They find that costly fragmentation can favor many more types of fragmentation modes than costless fragmentation. The three types of fragmentation cost have the same set of forbidden life cycles. Binary fragmentation modes are promoted for the case of costly fragmentation. If fragmentation costs occur for complexes of different sizes, then benefits of forming complexes of multiple cells are not essential for evolution of multicellularity. The authors argue that the three types of fragmentation costs that they consider are applicable to a broad range of species.

This article is well-researched, and the topic of costly fragmentation has relevance for understanding the life cycles of many organisms. I consider this paper a great conceptual achievement. I recomend publication subject to minor revision.

Thank you for a such a positive assessment of our work!

I have some minor comments:

It might be helpful to cite the following articles:

Ghang, W. & Nowak, M.A. (2014). Stochastic evolution of staying together. J. Theor. Biol. 360: 129-136.

Olejarz, J. W. & Nowak, M.A.(2014). Evolution of staying together in the context of diffusible public goods. J. Theor. Biol. 360:1-12.

Kaveh K, Veller C, Nowak MA. Games of multicellularity. Journal of Theoretical Biology. 2016;403:143–158

Olejarz, J., Kaveh, K., Veller, C. & Nowak, M.A. (2018). Selection for synchronized cell division in simple multicellular organisms. J. Theor. Biol. 457.

Note also that the model for evolution of eusociality in Nowak, M. A., Tarnita, C. E. & Wilson, E. O. (2010). The evolution of eusociality. Nature 466(7310):1057-1062
is a mathematical analysis of life cycles with costly fragmentation. It might be useful to point out that very interesting connection.

Thank you for highlighting these interesting papers. We added references to them.

page 5, middle: typo: "by dividing cell staying together" should be "by dividing cells staying together"

Thank you for such a careful reading of our manuscript. This and all typos below are fixed.

page 5, bottom: this might read better: "Units of size $i$ have death rate $d_i$, and cells in such a unit have division rate $b_i$."

page 6, middle: a comma is missing: "A partition is a way of decomposing an integer m into a sum of integers without regard to order, and summands are called parts [Andrews, 1998]."

page 7, middle: typo: "maximises" should be "maximizes"

page 7, bottom: typo: "where T it the fragmentation delay" should be "where T is the fragmentation delay"

page 8, caption of Figure 2: typo: "and remaining four are split" should be "and the remaining four are split"

page 11, top: typo: "see Appendix A.2 for details of the matrix construction." should be "(See Appendix A.2 for details of the matrix construction.)"

page 13, caption of Figure 3: typo: "and is thus called forbidden" should be "and are thus called forbidden"

page 16, caption of Figure 4: typo: "Fragmentation by binary and (nearly) equal split partitions are likely to evolve in random environments." should be "Fragmentations by binary and (nearly) equal split partitions are likely to evolve in random environments."

page 18, caption of Figure 5: typo: "found her have" should be "found have"

page 19, bottom: typo: "these modes delivering" should be "those modes delivering"

page 21, middle: typo: "both daughter cell" should be "both daughter cells"

page 21, middle: typo: "leaving empty maternal cell wall behind" should be "leaving the empty maternal cell wall behind"

page 22, top: typo: "cell give rises to the a colony" should be "cell gives rise to a colony"

page 22, bottom: typo: "which lead to the formation" should be "which leads to the formation"

page 23, top: typo: "which make them stick together" should be "which made them stick together"

page 23, middle: "Which of two" should be "Which of those two options"

page 23: The last paragraph should be either deleted or rewritten.

We decided to remove this paragraph entirely.

page 7, top: "Next, we assume that the fragmentation of a unit comes at a cost for this unit. In other words, if the unit continues to increase in size instead of fragmenting, its expected net biomass in the short run will be larger." Could that statement be clarified?

Sorry for being unclear here. We have now reformulated this sentence.

Reviewer #2: The paper is a theoretical investigation of how simple multicellular organisms evolve. The number of cells in these multicellular units (stochastically) grows and upon reaching a certain size, these units fragment into smaller units. The authors are interested in the most efficient way to do this. Or rather, the effects of the cost of fragmentation on the most efficient way. In order to do this they study rate equations describing the mean behavior.

My problem with the paper is that it falls somewhere between useful and beautiful. It is very far from experimental testing, nothing is known, so many parameters are assumed. To get answers one needs to know these parameters. As they write: "Unfortunately, such experimental data is not available, neither for natural nor for experimental populations."

Thank you, indeed our model has a number of parameters that we do not fit to a concrete biological system – birth and death rates at each intermediary group size. However, these parameters are the typical components of any demographic model. The entire field of demography is based on the same parameters and assumptions and this field greatly contributes to understanding of population dynamics in plants, vertebrates, and especially humans. However, this class of models is so far not routinely applied to microbial species and the evolution of multicellularity. Therefore, the experimental data are not readily available. Nevertheless, the methodology to gather such data exists and is well developed. Once a sub-field of the demography of simple multicellular species will emerge, our model can naturally be adapted to the new experimental paradigm. Predictions from our model are ready to be tested once experimental data are available, but we are not experimentalists and not in a position to conduct such experiments (as probably most mathematical biologists). In the revised manuscript, we reflect this aspect of our work in the discussion.

Hence the authors assume random parameters from some arbitrary distributions. And the results of course cannot be compared to data either.

First of all, we would like to stress that many of our analytical results are valid for any parameter values, e.g. Fig. 3 does not rely on concrete parameters. Our random parameter exploration is just one component of our work. We agree that individually, the analysis of each individual random profile does not contribute much. However, the random parameter values we use are not intended to replace unavailable experimental data. Even if experimental data for one or even many species were available, our present findings would have had an independent value – as it is an investigation into the multidimensional parameters space as a whole - wich will be hardly possible even once data becomes available. The set of random profiles is designed to provide an extremely diverse manifold of parameters values – may be even more diverse than the natural birth and death rates profiles. Hence, it seems safe to assume that the collection of life cycles observed in our study is at least as diverse as the one expected from natural populations, assuming that our model meaningfully captures the main aspects of life cycle evolution in nature. Yet, we found that the observed life cycles set is extremely biased towards binary fragmentation and equal split of groups. Our review of the natural life cycles demonstrates the same bias – these two classes of life cycles are overrepresented in the known natural populations as well. In our revised manuscript, we discuss this topic in more details.

And this would be OK if the model was pretty, which is subjective of course, but I find it cumbersome. The rules are quite arbitrary; one could similarly set up models with very different rules, like fragmenting at some rate at each size, fragmenting randomly, and so on.

Thank you for highlighting this. While we present the competition between a discrete set of life cycles, the general scenario you described is nevertheless taken into account in full extent in our manuscript. We can imagine a scenario, where at each size, a group may fragment by any available partition with some probability, or stay together. If the fragmentation does not happen, the group will grow larger – and will be subjected to stochastic fragmentation again. This model was considered in a previous paper (Pichugin et al, PLOS CB, 2017) and we found that in a such general model, the evolutionarily optimal life cycle must be deterministic for any parameters choice, i.e. the probability of a single fragmentation partition is equal to one, while probabilities of all others are zero. This result holds true in our current work as we demonstrate in appendix A.6. We capitalized on it, consider only deterministic life cycles in the main text.

The authors already published a conceptual paper Pichugin et al 2017, building upon Tarnita et al 2013. That paper made the point that there could be optimal fragmentation modes, and suggested that "in the field and in the laboratory" this could be studied. Last year a stochastic version of the model was published: Pichugin et al 2019.

We admit that this manuscript is part of a research line that we are pursuing. In 2017, we published first the approach, in 2019 we analyzed interacting cells and changing environments. In the current manuscript, we highlight a new aspect arising from our continued reading of the experimental literature, which suggests that fragmentation is not costless, as we assumed in earlier work.

In summary, I am not convinced that different versions and aspects of this model is worth being published in this Journal without meaningful links to biology.

Here we politely disagree, as our work suggests evolutionary foundations of the emergence of multiple fragmentation life cycles, which are quite common in nature. To our knowledge, this question has not been answered yet. Also, we present an example of the evolution of multicellular life cycles without any ongoing benefits to the cell group, which is, again, a possibility which was not even considered in the field before. We would like to attract your attention to our discussion of natural populations with respect to our assumption of costly fragmentation, especially the patterns of reproduction observed in *Nannochloris* genus.
