# Supplementary material for: Evolution of multicellular life cycles under costly fragmentation
Source: PLoS Comput Biol. 2020 Nov 19;16(11):e1008406. doi: 10.1371/journal.pcbi.1008406 (PMC7714367; doi:10.1371/journal.pcbi.1008406)
Supplement: Attachment — Submitted filename: Reply Rev 2_v4_AT.docx [file pcbi.1008406.s008.docx]

Dear Dr. Pichugin,

Thank you very much for submitting your manuscript "Evolution of multicellular life cycles under costly fragmentation" for consideration at PLOS Computational Biology. As with all papers reviewed by the journal, your manuscript was reviewed by members of the editorial board and by several independent reviewers. As Reviewer 1 had only minor concerns but Reviewer 2 recommended Reject we sent it back to Reviewer 2. Unfortunately, despite several reminders, Reviewer 2 did not respond, so we asked a third Reviewer to assess the whole situation given the previous reviewers. This reviewer is very positive but raises some issues. We would be very happy to consider a revised version that takes this comments into account.

Sincerely,

Philip K Maini
Associate Editor
PLOS Computational Biology

Natalia Komarova
Deputy Editor
PLOS Computational Biology

Thank you for handling our manuscript. We appreciate the comments given by Dr. Constable and incorporate them in the revised version of the manuscript. We explain the changes introduced in the new version next to each of the reviewers comments below.

We would like to add that one of the suggestions brought by Dr. Constable allowed us to find an error in our simulations. In the detrimental environments scenario, the cell birth rates profiles were sorted by the value ib_i_, while they should have been sorted by b_i_. We fixed our code and updated the affected figure 5. This leads to minor changes in the figure. The most important of one is that the effect of risk costs on the evolution of multicellular life cycles is much stronger than we had reported before. All interpretations of this figure presented in the paper remained valid.

Reviewer's Responses to Questions

**Comments to the Authors:
Please note here if the review is uploaded as an attachment.**

Reviewer #3: Review: Evolution of multicellular life cycles under costly fragmentation

The authors present an an analysis of how costs associated with the fragmentation of multicellular complexes may drive the evolution of particular fragmentation modes – for instance whether to cleave in to two equally sized parts, produce unicellular propagules or spilt into a number of unicells. The identification of conditions that lead these latter two results are particularly interesting, arising as they do thorough extensions of the authors’ previous 2017 work, where fragmentation costs were only briefly (and incompletely) touched upon. In the conclusion in particular, I believe the authors put together a solid case that different fragmentation costs could lie behind the variety of fragmentation modes observed in nature. From a theoretical standpoint, I also found the observation that these costs could actually drive the evolution of multicellularity itself very intriguing. On first reading I felt like this was likely be a purely theoretical curiosity. However following the authors’ discussion of reproduction in the Volvocales algae, I’m actually thoughtful of paying this more heed.

I think this is a thoughtful and thorough article, well-worthy of publication in PLOS Computational Biology. As always however, I believe there are some changes that the authors could make to improve the manuscript still further.

Thank you for such a positive assessment of our work and the thoughtful comments below that improved the exposition of our results.

The initial three suggestions I believe in some sense may address the concerns of Reviewer 2.

First, for the random environments the authors choose b_i and d_i sampled independently from the uniform distribution U(0,1). As Reviewer 2 states “Hence the authors assume random parameters from some arbitrary distributions. And the results of course cannot be compared to data either.” While I do not share Reviewer 2’s concern about data in a quantitative sense, it seems more biologically relevant to consider three subcases for b_i; b_i strictly increasing, b_i strictly decreasing, and a unimodal distribution for b_i (and simultaneously the converse scenario for d_i) . Importantly, it seems biologically unlikely that b and d are multimodal, as is possible in the current implementation.

The authors have already considered the second of these scenarios in Section 4.5, so there’s no extra work there. The case of strictly increasing b_i should be similarly straightforward. My guess would be that in this condition the “Other” fragmentation modes would be unlikely. The case of unimodal b_i would still allow for a formidable parameter space. A manageable subcase to look at might be b peaked in the middle at b_10. My guess here would be that we might see an increase in the maturity size selected for compared to the case with no costs, similar to the current results in Section 4.5.

Thank you for a very nice suggestion. In the revised manuscript, we now consider two additional cases: performance improving with size (b_i goes up with size, d_i goes down) and unimodal performance (there is an optimal colony size – 10 cells as you suggested - with the largest b_i and smallest d_i, colonies, while sizes further away from the optimal one have worse performance). There, we found that the scenarios of delay and loss are dominated by binary fragmentation with an addition of (nearly) equal split life cycles. The case of risk costs provided greater an advantage to (nearly) equal split and rarely allowed some “other” life cycles to emerge.

As for the changes in maturity size with increased costs, your intuition is absolutely correct for costs caused by loss: the more cells are lost, the larger units become. However, this is not true for the other two implementation of costs. For example, an analysis of our equations for the fragmentation with delay reveals that the maturity size under large delay is determined by the minimum of the death rate (*d_i_*). If fragmentation is delayed such that a multicellular unit spends a long time at the maturity state, the primary selection pressure is to stay alive during maturity, so the most protected state becomes the optimal maturity size. This state is different between different scenarios of b_i_/d_i_ profiles. In the true random scenario, this location is evenly distributed between sizes 1 and 19. In the beneficial scenario, it is the maximal available size - 19, in the unimodal scenario, it is the optimum size – 10, finally, in the detrimental scenario, the most protected state is a solitary cell. Consequently, the changes in costs result in diverse patterns of maturity size change. For the case of costs by risk, the similar analysis only restricts the minimal possible maturity size. The observed patterns of the maturity size are again diverse. As this issue becomes very complicated for these scenarios, we think that a detailed analysis of this goes beyond the current scope of our paper.

Second, in response to Reviewer 2’s concerns about data, the authors state “Predictions from our model are ready to be tested once experimental data are available, but we are not experimentalists and not in a position to conduct such experiments (as probably most mathematical biologists). In the revised manuscript, we reflect this aspect of our work in the discussion”. I believe the discussion tackles this very well, but suggest that the predictions of the model could be even more succinctly stated for use by experimentalists by using a table. This could be included in addition to the discussion (which could be kept as is) and could essentially list each species described by the authors, its empirically observed fragmentation mode, and the model predictions for scenarios under which this mode might be selected for. The table would then serve as a handy reference for experimentalists interested in testing these hypotheses.

Indeed, to have a summary table would help readers a lot. We have now implemented this nice suggestion.

Thirdly, Reviewer 2 is concerned that the current paper is perhaps incremental “The authors already published a conceptual paper Pichugin et al 2017 … I am not convinced that different versions and aspects of this model is worth being published in this Journal ...”. Given this it appears that it may not be emphasised clearly enough which predictions of the current model are novel with respect to the 2017 paper. I would suggest periodic reminders in the results section. To avoid repetition I would move the line 267-268 “Only binary fragmentations can be evolutionarily optimal under costless fragmentation, T = R = L = 0 [Pichugin et al., 2017]” (it’s not necessary to address the evolutionary dynamics in that section) and place it in a paragraph before L316 noting that nearly equal split, seeding, and other are not possible in the costless case. The following paragraphs could then emphasise how each fragmentation cost extends the life-cycles of the costless case – e.g. following line 321 “Thus delay fragmentation costs extend the possible life cycles to equal splits relative to the costless case”.

We agree that it is a beneficial change and highlighted the new findings observed in the present study.

My other comments are more minor/unrelated to previous reviews.

5) I was surprised to see discussion of the unicellular bottleneck omitted. I am not suggesting that this should be dealt with in a modelling sense, but it certainly should be discussed. It is likely that this is a strong factor driving a transition from equal-part fragmentation modes to those involving the production of unicells.

We have added a paragraph on the single cell bottlenecks to the discussion.

6) Section 3.2 could be improved by explicitly giving an example with a given \pi(\kappa) and m, and emphasising these as evolutionary parameters. This might also help make the life-cycle definitions (e.g. 1+1 lifecycle) more clear.

Thank you for attracting our attention to this section. We improved our explanation of fragmentation modes. We added the explanation of m and reference to fig 2B illustrating the scenario we discuss. We removed the additional case of fragmentation with a single cell dying, as reproduction costs are only introduced in the next section. We changed the maturity size considered from m=2 to m=3 to demonstrate more examples. However, we do not think that it would be beneficial to show \pi(\kappa) values here. These quantities are not needed until section 3.5, where a detailed example is given.

7) L149 - “The fragmentation cost is another external factor of our model” - would it be more natural to think of this as a physiological restriction, based on the evolutionary history of the organism under consideration?

We agree that it is much better exposition of the fragmentation costs. We changed the this part of the manuscript as you suggested.

8) Fig 2 could reference Eqs.(1-2) for the b/d costs.

Done.

9) Figure 3 – in the “nearly equal split”, 1+1+1+…1 is included, yet this seems by far the more interesting example in this category biologically, and it also appears in particular under various scenarios. Would it be worth considering this as a separate case?

We want to state that we like you suggestion a lot and also very curious about multiple fission life cycles 1+…+1. However, the focus of our manuscript is on the investigation of the effects of various mechanisms of reproduction costs and broad characterization of possible life cycles. Investigation into the details of (even very interesting) cases would be a detour from the current direction. Also, even in the present form, the manuscript seems rather heavy in content and concepts, so adding more ideas will harm readability. We hope we can study these and other life cycles abundant in nature in our future work on this topic.

10) L133 fast → quickly

Done.

Thanks a lot once more!
